# Dredging and sediment discharge in a river with floodplain. Physicochemical and microbiological effects in Paraná river

**DOI:** 10.1016/j.heliyon.2024.e41224

**Published:** 2024-12-17

**Authors:** Maria Josefina Gonzalez, Stella Maris Gonzalez, Aldo Raul Paira, Pablo Agustín Collins

**Affiliations:** a Instituto Nacional de Limnología (CONICET-UNL), Ciudad Universitaria Pje. El Pozo s/n Santa Fe, CP 3000, Argentina; b Facultad de Ingeniería y Ciencias Hídricas – Universidad Nacional del Litoral, Ciudad Universitaria Pje El Pozo s/n Santa Fe, CP 3000, Argentina; c Facultad de Bioquímica y Ciencias Biológicas – Universidad Nacional del Litoral, Ciudad Universitaria Pje El Pozo s/n Santa Fe, CP 3000, Argentina; d Centro Operativo Experimental A.G. (EEA Rafaela - INTA), Angel Gallardo s/n, CP 3014, Argentina

**Keywords:** Dredging, Paraná river, Heavy metals, Nutrients, Bacteria communities

## Abstract

Alterations caused by human activities in the environment, such as dredging, modify the physicochemical conditions and affect the habitat. Maintenance dredging that allows large vessels access to inland ports is a recurring disruptive action. The study aimed to evaluate, during a maintenance dredging operation in a port area of the Paraná River, the modifications in the structure of the river, the presence of contaminants and bacterial organisms. The effects caused during the operation of a suction dredge in a South America river under two temporal conditions were analyzed: i) the short and medium-term effects on the physicochemical variables, and ii) the immediate effects on the physicochemical variables and the abundance of bacterioplankton. The variables measured in sediment and water samples were limnological parameters (e.g. conductivity, pH, dissolved oxygen, among others), heavy metal concentration, presence of biocides, hydrocarbon molecules, nutrients, sediment granulometry, *Escherichia coli*, *Enterococcus*, total coliforms, and heterotrophic bacteria. Some physicochemical variables increased in the water column immediately after the water mass passed through the operating dredge, including sediment resuspension. The parameter changes were transient, as there were no significant increases in the variables downstream of the dredge during dredging and discharge operations or after dredging work. Some metal concentrations increased in the sediment and water column. Bacteria increased during dredging and more after rainfall events. Then, at the end of the dredging, the bacteria concentrations decreased to previous values. The possible effects of dredging disturbance were of the same order or less than those of natural ones, i.e. rainfall. Sixty days after the dredging work was completed, the system was back to normal both in the dredged and discharge areas.

## Graphical abstract

**Figure:**
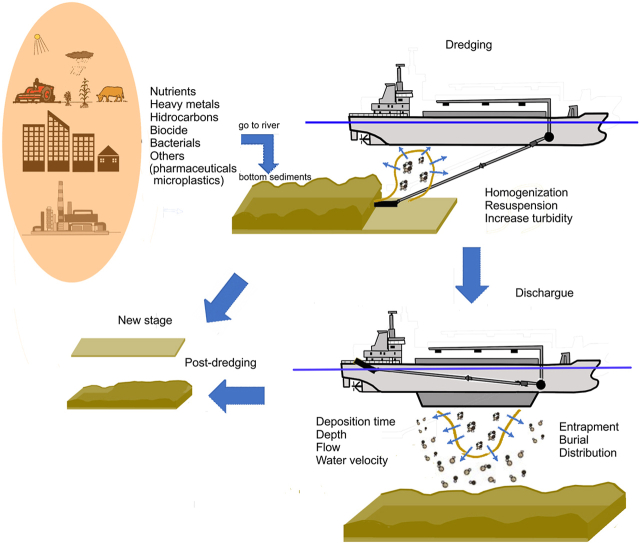

## 1 Introduction

Aquatic environments are under anthropic pressure for different reasons, including urbanization, industrialization, and progressive changes in land use. These cause morphological, physicochemical and biological alterations of habitats [[1], [2], [3]]. Among them, contamination from point and nonpoint sources, such as municipal wastewater and storm drains, affect environmental conditions. These discharges carry microbial pollution, among other contaminants, especially near large population centers, industries, or animal breeding establishments [[4], [5], [6]]. Biological contamination is mainly composed of bacteria of human and animal origins as a result of the dumping of untreated wastewater directly into rivers and the dragging of animal feces caused by runoff during rains. This causes one of the most serious public health problems worldwide, namely gastrointestinal and respiratory infections [7,8].

In addition, heavy metals, biocides, persistent organic pollutants (POPs), nutrients, and other chemicals (e.g. detergent, oil-derived) are carried by runoff into the river and travel downstream or accumulate in the river sediment [[4], [5], [6], [7], [8], [9], [10], [11], [12]]. Their accumulation in sediment beds becomes potential sources of contamination for the present and the future [13]. Furthermore, dissolved organic and inorganic contaminants in the environment can be adsorbed by suspended sediment particles and then, through deposition, form contaminant reservoirs in the bottom sediments [14]. Many contaminants, such as hydrophobic organics (e.g., PCBs) and some inorganics, tend to remain strongly adsorbed to sediments even after mechanical resuspension in the water column [15]. Nutrients, nitrogen and phosphorus, compounds also bind to sediments and become immobilized [16]. The mechanism of immobilization of nutrients and contaminants by sediments involves, among others, absorption at ion exchange sites, binding to organic matter, incorporation into reticular structures, and precipitation into insoluble compounds [16]. Contaminants and organic matter show affinities for the finer fraction of aquatic sediments. The exponential increase in surface area with decreasing sediment grain size and increases in surface charge justify the best associations [[17], [18], [19]].

The constant deposition of sediment on the river beds [20] combined with the need to have commercial and navigable routes between countries forces port areas to carry out dredging activities. Dredging of navigation channels for ship access to port as a maintenance action is necessary to ensure adequate depth to meet the safety and maneuverability requirements of ships. The growth of world trade causes an increase in the number of ships and their capacities and sizes, which therefore require extensive dredging services to reach port areas [21].

Dredging has three main stages, extraction, transport, and disposal. Different types of dredgers can be used, from cutter suction dredgers to trailer suction hopper dredgers [22]. Dredging operations in rivers include an increase turbidity and suspended sediment with changes in water quality and conditions to aquatic life [22]. It involves the resuspension of sediment-bound bacteria with fecal and other enteric pathogens [23].

The Paraná River has a hydrographic basin that covers a large part of south-central South America. It is the second largest on the continent, only surpassed by the Amazon River. Its delta is a huge swampy and forested area. The lower and middle Paraná is navigable and is used as a waterway connecting inland cities with the Atlantic Ocean. Large ocean-going vessels can navigate up the Paraná River as far as the cities of Santa Fe or Paraná, but beyond that point, only small river vessels can reach Paraguay and Brazil [24]. The heterogeneity and dynamics of the environmental characteristics of the Paraná River allow the development of biological communities that inhabit these ecosystems [25]. Therefore, alterations in this mosaic of environments that occur due to anthropogenic disturbances could be difficult to distinguish from natural changes. Achieving an accurate assessment of anthropogenic impacts is necessary to improve the management of river development while maintaining a balance with a ‘good ecological status’ of the aquatic environment [26].

For several decades, studies of the dredging process have interpreted the effect of released contaminants as metals or hydrophobic organic pollutants [27]. Furthermore, they investigated the effects of fish at different stages of their life [28], and others evaluated the effect of dredging sounds [29]. Some of them are related to dredging activity, sediment resuspension, and bacterial communities. However, these studies were not carried out on the Paraná River, the second largest river in South America.

The study aimed to evaluate, during a maintenance dredging operation in a port area of the Paraná River, the changes in the structure of the river, the presence of contaminants, nutrients, and bacterial organisms. Two different temporal samplings were proposed. i) Analyze the short- and medium-term cumulative effects of a maintenance dredging operation on the physicochemical variables and contaminants. ii) Analyze the immediate effects of a suction dredge on the physiochemical variables and bacterioplankton. These objectives are designed taking into account the resuspension and morphological modification processes that dredging causes on the river and its bed.

## 2 Material and methods

### 2.1 Description of the dredging area

The Paraná River is the main lotic system of the Plata Basin. It represents the most important navigable route through which products from north-central Argentina, Paraguay and Bolivia are shipped to countries in Europe, Asia, Africa and America. The lower and middle sections of the Paraná River are characterized by a network of main and secondary channels, islands, and shallow internal lakes that form a large floodplain [30].

**Fig. 1:**
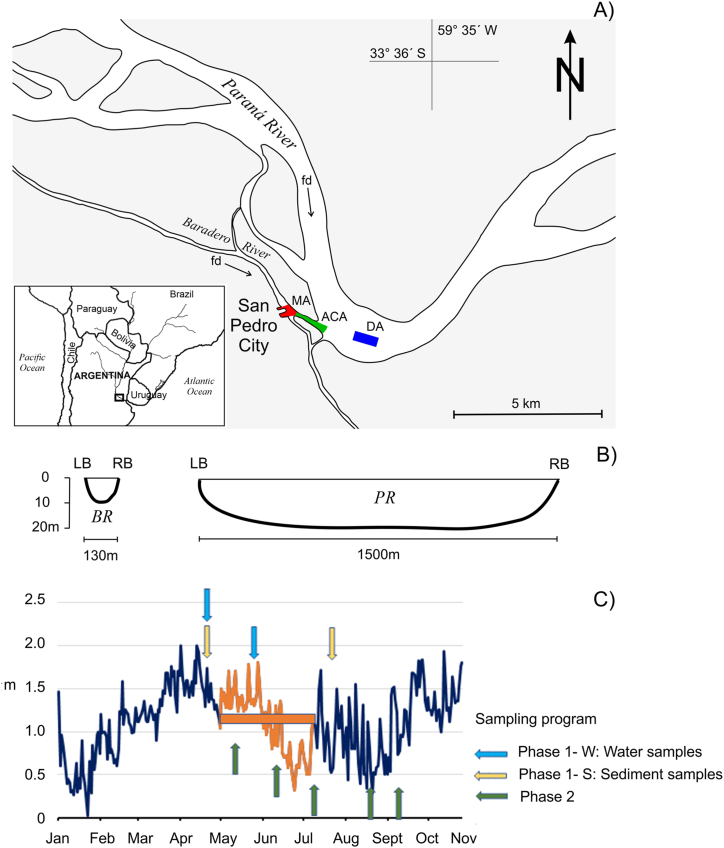
A) Paraná and Baradero Rivers near the San Pedro port showing study area (fd: flow direction, MA: maneuvering area, ACA: access channel area, and DA: discharge area), B) Bathymetric profile in the dredging area and discharge area (BR: Baradero River, PR: Paraná River, LB: left border, RB: right border), C) Height of the river in the Port of San Pedro, indicating the dredging period (orange line) and the sampling times (arrow) corresponding to both phases of study.

The study area corresponds to the port of the city of San Pedro (Argentina) (33°41'11'' S, 59°38'11'' W) on a secondary channel of the Paraná River named the Baradero River. The dredging area is approximately 1,500 m long and 120 m wide, including the access channel and maneuvering area. The dredged material is dumped into the main channel of the Paraná River over an approximate area of 700 m long and an average depth of 18 m (Fig. 1).

The climate of the region is temperate, although the river comes from warmer regions. The flow rate depends mainly on the rainfall in the upper basin of Paraná and Paraguay rivers, which generate a unimodal hidrosedimentological regimen. Furthermore, the Port of San Pedro is influenced by the tidal regimen due to its proximity to the mouth of the sea (Fig. 1). The morphology of the Paraná River in its middle and lower sections is a mosaic of wetlands. The main channel, secondary channels and shallow lakes are interconnected (Fig. 1) and, according to the water level, isolated from surrounding land areas. The navigable channel to reach the port of San Pedro requires an average depth of 10 m, which is dredged every one or two years to ensure this navigation depth. In the winter of 2023, a maintenance dredging operation was carried out in the navigable channel and maneuvering area of the port. The dredging work was carried out using a trailer suction dredger.

### 2.2 Field sampling program

The field sampling program consisted of two phases for the same dredging work in the port of San Pedro City. The first was carried out before, during, and after the dredging, while the second, the samplings were during the dredger-boat work and after dredging.

Independently of the sampling program, before, during, and after dredging, the morphological characterization of the river bottom in the dredging and discharge area is carried out using bathymetry.

The first phase was carried out at seven sites located in the dredging (5) and discharge (2) areas (Fig. 1; Table 1). Water samples were taken with a van Dorn bottle at 1 m of depth in the water column and sediment samples from the river bottom. Conductivity, pH, turbidity, dissolved oxygen, BOD5, COD, total nitrogen Kjeldhal, nitrates, nitrites, total phosphorus, suspended solids, total dissolved solids, hydrocarbons, heavy metals, phenols, cyanide, and total sulfides were measured in the water before the start of dredging and three weeks after the dredging process began (Table 2).

**Table 1:** Program of sampling with date of samples, locations, and name of sample sites used in the study of dredging at San Pedro Port, Argentina.

Phase 1- W: Water samples				Phase 1- S: Sediment samples				
Pre-Dredging, Date: 04/19/23 Dredging Date: 05/22/23				Pre-Dredging Date: 04/19/23 Post-Dredging Date: 07/19/23				
MA: Maneuvering Area			ACA: Access Chanel Area			DA: Discharge Area		
MA1	MA2	MA3	ACA1	ACA2		DA1	DA2	
33°41'13''S 59°38'6''W	33°41'12''S 59°38'22''W	33°41'7''S 59°38'19''W	33°41'32''S 59°37'30''W	33°41'21''S 59°37'47''W		33°41'53''S 59°36'37''W	33°41'51''S 59°36'58''W	
Phase 2								
Dredging: Date: DT1: 05/10/2023; DT2: 06/08/2023; DT3: 07/06/2023 Post-dredging: Date: PdT1: 08/17/2023, PdT2: 09/19/2023								
Dredging area					Discharge area			
UD1	UD2	**UB∗**	**DS∗**	DD	UDiS1	UDiS2	**DiS∗**	DDiS
33°40’00″S 59°40’22″W	33°41’12″S 59°38’45″W	33°41’10’’″S 59°38’16″W	33°41’12″S 59°38’10″W	33°41’36″S 59°37’28″W	30°50’60″S 59°37’37″W	33°41’36″S 59°37’28″W	33°41’48″S 59°36’42″W	33°41’56″S 59°30’05″W

UB∗, DS∗ and DiS∗: the dredger-vessel position is variable depending on the working area during sampling.

**Table 2:** Physical-chemicals parameter measured in the two phases of the study of dredging at the San Pedro Port, Paraná River, analysis methods and detection limit based according to Bouyoucos [32]; APHA [33]; EPA [34,[35], [36]].

Parameter	Analysis Method	Lim detection		Parameter	Analysis Method	Lim detection	
		Sediment	Water			Sediment	Water
Phase 1							
Silt	Hydrometer method	0.1 % p/p	–	Benzo (A) anthracene	EPA 8270D	0.01 mg kg^−1^	0.005 μg l^−1^
Clay		0.1 % p/p	–	Benzo (A) Pyrene	EPA 8270D	0.01 mg kg^−1^	0.005 μg l^−1^
Sand		0.1 % p/p	–	Benzo (B, K) Fluoranthene	EPA 8270D	0.01 mg kg^−1^	0.005 μg l^−1^
pH	Multiprobe	0.01	0.01	Benzo (G, H, I) Perylene	EPA 8270D	0.01 mg kg^−1^	0.005 μg l^−1^
Conductivity		–	1 μS cm^−1^	Chrysene	EPA 8270D	0.01 mg kg^−1^	0.005 μg l^−1^
Turbidity	SM 2130 B	–	0.5 NTU	Dibenzo (A, H) Anthracene	EPA 8270D	0.01 mg kg^−1^	0.005 μg l^−1^
Suspended Solid (SS)	SM 2540 D	–	1 mg l^−1^	Phenanthrene	EPA 8270D	0.01 mg kg^−1^	0.005 μg l^−1^
Total Dissolved Solids (TDS)	SM 2540 C	–	1 mg l^−1^	Fluoranthene	EPA 8270D	0.01 mg kg^−1^	0.005 μg l^−1^
Total Organic Carbon (TOC)	Walkley and Black	0.1 %	–	Fluorene	EPA 8270D	0.01 mg kg^−1^	0.005 μg l^−1^
Dissolved Oxygen (DO)	Multiprobe	–	1 mg l^−1^	Indene (1,2,3-CD) pyrene	EPA 8270D	0.01 mg kg^−1^	0.005 μg l^−1^
Biochemical Oxygen Demand (BOD)	SM 5210 D	–	1 mg l^−1^	Naphthalene	EPA 8270D	0.01 mg kg^−1^	0.005 μg l^−1^
Chemical Oxygen Demand (COD)	SM 5220 D	–	10 mg l^−1^	Pyrene	EPA 8270D	0.01 mg kg^−1^	0.005 μg l^−1^
Total Nitrogen (TKN)	SM 4500 NORG D	–	0.6 mg l^−1^	Biocides			
Nitrate	SM 4500 NO3∼ D	–	0.1 mg l^−1^	Chlorpyrifos	EPA 8141B	0.1 mg kg^−1^	–
Nitrite	SM 4500 NO2∼ B	–	0.01 mg l^−1^	Dichlorvos	EPA 8141B	0.1 mg kg^−1^	–
Total Phosphorus (TP)	SM 4500 P B/E	–	0.01 mg l^−1^	Mevinphose	EPA 8141B	0.1 mg kg^−1^	–
Total Cyanide (TC)	SM 4500 CN F	–	0.005 mg l^−1^	Azinphos methyl (Guthion)	EPA 8141B	0.1 mg kg^−1^	–
Total Sulfide (TS)	SM 4500 S2- F	–	0.1 mg l^−1^	Parathion methyl	EPA 8141B	0.1 mg kg^−1^	–
Phenols	SM 5530 B	0.5 mg kg^−1^	0.01 μg l^−1^	Malathion	EPA 8141B	0.1 mg kg^−1^	–
Oil and fats	Gravimetric	2 2 mg kg^−1^	–	Parathion	EPA 8141B	0.1 mg kg^−1^	–
Heavy metals				Dimethoate	EPA 8141B	0.1 mg kg^−1^	–
Cadmium	EPA 7130	0.05 mg kg^−1^	0.1 μg l^−1^	Alpha-Hexaclorocyclohexane	EPA 8081B	0.01 mg kg^−1^	–
Total Copper	EPA 7210	2 mg kg^−1^	0.02 μg l^−1^	Methoxychlorine	EPA 8081B	0.01 mg kg^−1^	–
Total Chromium	EPA 7190	5 mg kg^−1^	0.05 μg l^−1^	Aldrin	EPA 8081B	0.01 mg kg^−1^	–
Total Nickel	EPA 7520	5 mg kg^−1^	0.05 μg l^−1^	Dieldrin	EPA 8081B	0.01 mg kg^−1^	–
Lead	EPA 7420	5 mg kg^−1^	0.05 μg l^−1^	Endrin	EPA 8081B	0.01 mg kg^−1^	–
Total Zinc	EPA 7950	5 mg kg^−1^	0.05 μg l^−1^	Endosulfan I	EPA 8081B	0.01 mg kg^−1^	–
Arsenic	EPA 7061A	0.1 mg kg^−1^	5 μg l^−1^	Endosulfan II	EPA 8081B	0.01 mg kg^−1^	–
Total Mercury	EPA 7470A	0.01 mg kg^−1^	0.1 μg l^−1^	Endosulfan Sulfate	EPA 8081B	0.01 mg kg^−1^	–
Hydrocarbons				γ -BHC (Lindane)	EPA 8081B	0.01 mg kg^−1^	–
Total petroleum hydrocarbons (TPH)	EPA 418.1	1 mg kg^−1^	0.1 mg l^−1^	Methoxychlor	EPA 8081B	0.01 mg kg^−1^	–
Acenaphthene	EPA 8270D	0.01 mg kg^−1^	0.005 μg l^−1^	4,4′-DDE	EPA 8081B	0.01 mg kg^−1^	–
Acenaphthylene	EPA 8270D	0.01 mg kg^−1^	0.005 μg l^−1^	Heptachlor	EPA 8081B	0.01 mg kg^−1^	–
Anthracene	EPA 8270D	0.01 mg kg^−1^	0.005 μg l^−1^	Heptachlor Epoxide	EPA 8081B	0.01 mg/kg	–
Phase 2							
Temperature	Multiprobe HACH HQ40D	–	0.1 °C	Transparency	Secchi disc	–	0.1 cm
Conductivity		–	1 μS cm^−1^	Silt	Hydrometer method	0.1 % p/p	0.1 % p/p
ClNa		–	0.1 mg l^−1^	Clay		0.1 % p/p	0.1 % p/p
Dissolved Oxygen (DO)		–	1 mg l^−1^	Sand		0.1 % p/p	0.1 % p/p
pH		–	0.01	Organic Matter	Calcination and gravimetry	0.1 % p/p	0.1 % p/p

Bottom sediment samples were taken with a Beckman dredge before the dredging process and 10 days after the dredging work was completed. From these samples, the percentage of sand, silt and clay, pH, hydrocarbons, heavy metals, phenols, oils and fats, total organic carbon (TOC) and biocides were determined (Table 2).

The sampling sites were separated and grouped into maneuvering area (MA) (three sites), access channel area (ACA) (two sites), and discharge area (DA) (two sites) (Fig. 1, Table 1).

The second phase consisted of taking limnological parameters and microbiological samples at five sites associated with the dredging area (Table 1, Table 2). The sites were upstream of the dredging area (UD), upstream of the bow (UB) and downstream of the stern of the dredging vessel (DS), and downstream of the dredge area (DD) where the vessel was working. In addition, four samples were taken related to the discharge process (two sites upstream: UDiS1 and UDiS2), below it during the discharge process (DiS), and downstream of them (DDiS)) (Table 1).

Limnological variables and bacteriological samples were taken with multiprobes and microbiological bottles, respectively, at 1 m depth of the water column. The second phase was carried out three times during the dredging stage with an interval of approximately one month between each one. Two samples were then taken after the dredging process, approximately 30 and 60 days after the dredging was completed. Post-dredging sampling, since the dredging vessel was no longer working there, was carried out in the dredging and discharge areas. In addition, rainfall events and the volume of water falling in the region were recorded (https://ruralecz.com.ar/informedelluvias.html (accessed August 13, 2024)).

### 2.3 Morphometric analysis of the bottom

Bathymetric was performed with an echo sounder of two channels (Kongsberg EA440) and 50 m transects were traveled with an equidistant point distance of 12.5 m between each transect, during the pre-dredging, dredging and post-dredging.

### 2.4 Physicochemical analysis

Phase 1 samples were taken cold (4 °C) to the laboratory and analyzed using standardized methods (Table 2). The pH/ISE/Cond/OD Thermo Orion Star 5 multiprobe was used to analyze pH, conductivity, and oxygen dissolved. Gravimetry methods were performed using an analytical balance (Ohaus Pioneer PA214). The BOD was obtained in an incubator INGELAB I-316-D and the digestor to COD was HACH LTV02. Total nitrogen was measured with a BIOTEC Z-100 semi-automatic Kjeldhal distiller and a SHIMADZU 2101-PC UV–Visible spectrophotometer, as well as with other colorimeter methods (nutrient, sulfide, phenols). Selective ion analyses, such as nitrate and cyanide, were performed using an ion chromatograph (SHIMADZU PROMINENCE). Turbidity was measured with a Thermo Orion Aquafast II turbidimeter. An atomic absorption spectrophotometer (SHIMADZU AA-7000) was used for the analysis of metal concentrations and a HP 5890 Series II-FID gas chromatograph was used to measure hydrocarbons and biocides.

Calibration and Quality Control (QC) solutions were prepared from standard solutions (e.g. Merck and LGC Standards GmbH). Recoveries ranged from 89.2 to 109.4 % for all metals. The differences in metal concentrations between analyzed and certified values were less than 10 %.

Total suspended solids (TSS) concentration was measured from water previously filtered through a pre-combusted 0.7 μm pore filter (4 h, 500 °C) (Whatman GF/F). The filters were then dried (24 h, 60 °C) and weighed. Suspended organic matter (SOM) and inorganic matter (SIM) were obtained as weight loss by ignition (500 °C, 4 h). Nutrients, sulfide and cyanide concentrations were determined in filtered (0.7 μm) water samples. The accuracy of each parameter is given in Table 2.

The measurement of the parameters in the second sampling phase was carried out “in situ”, except for the granulometry of particulate and organic materials in sediment and in suspension (Table 2). The Hach HQD40 multiprobe was used to measure conductivity, NaCl, dissolved oxygen, oxygen saturation, pH and temperature.

Transparency was measured with a Secchi disk. The depth was recorded with a Konigsberg EA440 echosound.

Bottom sediment samples were taken with a Tamura dredge. The clays, silts and sands were separated by sieving. The suspended sediment was taken at 1 m from the surface with a 10-L collector and the water was trespassed to a 5-L bottle. The concentration of coarse sediment (very fine sand: >62.5 μm) and fine sediment (silt and clay: <62.5 μm) at the bottom and in suspension was determined after filtration. Organic matter was calculated by weight differences after calcination between the weight of inorganic and organic matter [31].

### 2.5 Bacteriological analysis

Surface samples were collected with sterile Simax glass bottles (500 ml), at 15 cm depth and kept refrigerated and in the dark until their analyses [37].

The concentration of *Escherichia coli* (*E. coli*), total coliforms (TC) and *Enterococcus* was used as an indicator of bacteriological quality. The group of heterotrophic bacteria (Het) was recorded for its role as an indicator of contamination, associated with the presence of autochthonous or allochthonous organic matter.

For bacteriological analysis, the plate count method was used. All samples were seeded by direct seeding or with the membrane filter technique (pore size of 0.45 μm) depending on the conditions of the samples.

To isolate and enumerate *E. coli* and TC, the enzymatic detection method (β-D-Glucuronidase) was used with a selective culture medium, Petrifilm™ EC/TC (3M™). To detect *Enterococcus*. KF-*Streptococcus* Agar with TTC (Triphenyltetrazoliumchloride) Solution 1 % (35 ± 2 °C for 46–48 h) was used [37]. To enumerate Het, 3M™ Petrifilm™ Aerobic Count Plate (35 ± 2 °C for 48 h) was used. The plates were incubated at different temperatures depending on the group: 44.5 + 0.5 °C for *E*. *coli* (24 h), 37 + 0.5 °C for Het, *Enterococcus* and TC (48 h) [37].

To assess the bacteriological quality of the water, the standard levels for primary contact with water established by recognized organizations such as the World Health Organization [7], the European Union [38] and the United States Environmental Protection Agency [39] were considered.

### 2.6 Data analysis

Data, where applicable, are presented as mean ± standard deviation (SD) and were first analyzed to assess normal distribution with Shapiro-Wilks tests. Then due to the failure of normality, Mann-Whitney U-tests were applied to compare two paired samples of the environmental and microbiological data sets and Kruskal-Wallis test to more than two samples. The threshold level of statistical significance was α = 0.05 for all analyses [40]. All experimental data were analyzed using the PAST 4.03 program [41].

## 3 Results

The dredging operation lasted 136 days (01-05-2023 to 07-07-2023), extracting approximately 250,000 m^3^ of fine sediments (silt and clay). The height level of the Paraná River in the port of San Pedro during the dredging operation ranged between 0.32 and 1.8 m, a period of minimum flow that corresponds to the climatic season of late autumn and early winter (Fig. 1). Water temperature had less variation in the dredging area and greater variation in the main channel of the Paraná River, but the differences were not significant (p = 0.97). The temperature range during dredging had a difference of 3.2 °C (17.3–20.5 °C) in the dredging area and the discharge area in the main channel. After dredging was completed, the temperature recorded lower but not different values (p = 0.06), varying between 16.5 and 17.5 °C in the dredging area and between 17.1 and 18.0 °C at the discharge into the main channel of Paraná River. In the days prior to the sampling of 5-10-23 and 8-17-23, rainfall between 22 and 109 mm respectively occurred in the region. In the third sampling (7-6-23), a storm occurred in a short period of time (34 mm in 30 min) producing intense drainage. While in the samples of 6-8-23 and 9-19-23 there is no precipitation.

### 3.1 Morphological analysis of the bottom

**Fig. 2:**
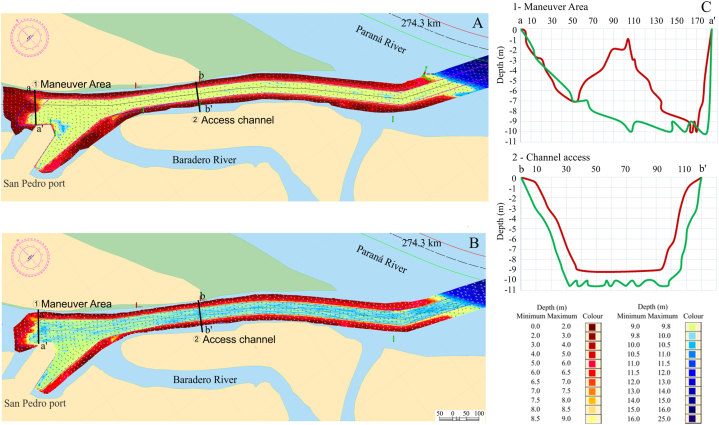
A) Pre-dredging, B) Post-dredging bathymetry of the study area (access channel and maneuvering area) of the San Pedro port- Paraná River, C) the bathymetric profile of two transects at the maneuvered area (a-a’) and access channel (b-b’) in pre-dredging (red line) and post-dredging (green line).

Dredging was carried out in the access channel and maneuvering area of the port of San Pedro. The first, approximately 1500 m long and 125 m wide with an area of 1.3 km2, had a minimum depth, before being dredged, of 8.6 m near the meeting point with the Paraná River and in the middle of the access channel a maximum depth of 9.6 m. Once the dredging was completed, the access channel had depths between 9.8 and 10.6 m along its length. The maneuver area has an approximate surface area of 0.12 km2 with minimum depths of 1.1 m and maximum depths of 10.0 m, reaching after dredging minimum and maximum depths of 8.0 and 10.3 m, respectively (Fig. 2).

The access channel has a smooth and low slope (0.004 %). The bottom of the channel has small undulations composed mainly of silt and clay. Dredging extracted up to 9 m of silt deposited in the ship dock area, but at the access channel, the removal corresponded to between 1 and 2 m of fine sediments, unifying the microforms (Fig. 2). In the dredge area, the percentage of silt increased, and the amount of clay decreased (before-dredged 47 % silt, 35 % clay, and 18 % sand; post-dredged: 67 % silt, 19 % clay, and 13 % sand).

The discharge area is located on the western margin of the thalweg of the Paraná River with an area of 700 m long by 100 m wide and depths between 18 and 27 m. The discharge did not affect the minimum and maximum depths of the area, having similar morphometry and depth between pre-dredging and post-dredging. However, there was a change in the composition of the sediments, from a silt:clay:sand ratio of 4:4:2 to 7:2:1 post-dredging.

### 3.2 Physicochemical analysis - phase 1

Some measured parameters varied depending on whether they corresponded to sediment or water samples and the proximity of the port and the drainage area of the city of San Pedro or the discharge area.

The Baradero River recorded conductivity values that did not vary between sites and sampling times, being 130.6 (3.57) μS cm^−1^ before dredging in the access channel and maneuvering area and during dredging activities 123.8 (9.85) μS cm^−1^. The discharge area also presented values at both times (pre-dredging: 128.8 (1.56) μS cm^−1^; dredging: 119.3 (0.92) μS cm^−1^).

**Fig. 3:**
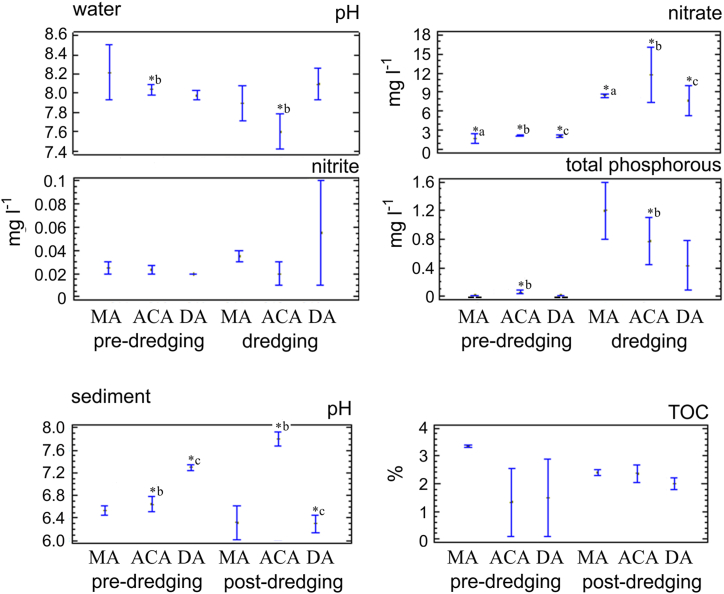
Mean ± standard deviation of some parameters measured in sediment and water at dredging area nearly to San Pedro Port (maneuvering area (MA) and access channel (ACA)), and discharge area (DA) at the Paraná River. ∗ with the letters a, b, and c indicate statistically significant differences between locations at the p < 0.05 level; - -: no detection.

The waters of both rivers, Baradero and Paraná, registered basic pH with variable values in the maneuvering area before the dredging work. Then, during dredging, a significant decrease in pH was only observed in the access channel (p = 0.009), while the maneuvering and discharge areas were similar (p = 0.19) (Fig. 3).

The sediments before dredging presented slightly acidic pH values, both in the samples from the access channel and in the maneuvering area. After the dredging work, a significant increase in pH was observed in the access channel (p < 0.001) and acidification in the discharge area (p = 0.015) (Fig. 3).

Dissolved oxygen was similar between dredging and discharge sites before and during the dredging procedure (dredged zone: 6.92 (0.239) ml l^−1^; discharge zone: 6.85 (0.071) mg l^−1^) (p = 0.73). In the same situation, BOD5 and COD values were similar between locations and times, with mean values of 2.8 (1.77) mg l^−1^ (p = 0.65) and 37.8 (7.01) mg l^−1^ (p = 0.85), respectively.

Total solid suspended (TSS) remained constant during dredging work in both areas (access channel and maneuvering area) (pre-dredging mean: 5.5 (4.28) mg l^−1^ and dredging 3.2 (2.11) mg l^−1^) (p = 0.74). An increase of TSS only occurred during discharge procedures (pre-dredging: 2.9 (0.13) mg l^−1^; dredging: 15.6 (17.82) mg l^−1^) (p < 0.001) (Appendix 1).

The TOC values were variable in the pre-dredging, being high in the area closest to the port and the city effluent discharge (maneuvering area). This indicator decreased after dredging and became similar in the access channel and maneuvering area (Fig. 3).

The detection and/or significant increase of nitrogenous elements and total phosphorus occurred in the access channel, maneuvering area, and discharge area between pre-dredging and dredging samples (p = 0.045). However, nitrite was similar between sites and time (p = 0.705), with highly variable values in the discharge area. Total nitrogen values by Kjeldahl remained constant at 7 mg l^−1^. Meanwhile, concentrations below the detection limit of cyanide and sulfides were indicated for all samples (Appendix 1).

**Fig. 4:**
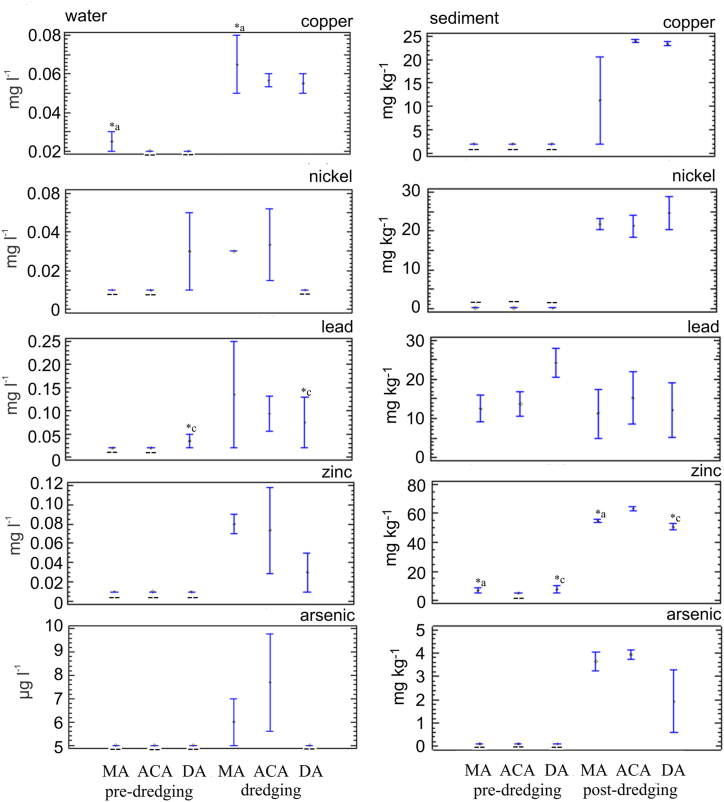
Mean ± standard deviation of some heavy metals measured in sediment and water at dredging area nearly to San Pedro Port (maneuvering area (MA) and access channel (ACA)), and discharge area (DA) in the Paraná River. ∗ with the letters a, b, and c indicate statistically significant differences between locations at the p < 0.05 level; - -: no detection.

Regarding the heavy metals analyzed, it is observed that the cooper increases significantly, or concentrations are detected in sediment and water samples from the post-dredging (p = 0.23) (Fig. 4). Although no cadmium was detected in the pre- and post-dredging sediment samples, a variable increase in the water column occurred in all areas with the highest value in the maneuvering area (MA1) during dredging. Chromium was present in the sediment and there were no significant differences pre- and post-dredging (p = 0.82), except in the discharge area (p = 0.02). The detection of this metal in the water column occurred at two specific points (pre-dredging: ACA 1; dredging DA2) with values close to the detection limit (Appendix 1).

Nickel increased in both, sediment and water samples, the latter with variable values. In the discharge area (DA), the highest content was observed only in the sediment, not in the water. Lead in the sediments of the dredging area (MA and ACA) recorded similar values in the pre- and post-dredging samples, and in the discharge area (p = 0.76). During the dredging works, the presence of Pb was observed in the water column (Fig. 4).

Zinc in the sediment and water samples increased significantly (p < 0.001; p = 0.01) between dredging and post-dredging. Concentrations during the first were variable in the water of the channel access.

The appearance of arsenic occurred in the sediment in the pos-dredging samples. While in the water column, it was observed in the channel access area (ACA) and maneuvering area (MA) in variable concentrations. It was not detected in the water samples from the discharge area during the dredging works (Fig. 4).

No mercury was recorded in sediment or water samples during pre-, dredging and post-dredging (Appendix 1). In all samples, hydrocarbons were not found (Appendix 1) or are below the detection limit.

The list of biocides selected, either due to their persistence and historical use and/or greater frequency of use today as well as their dangerousness, were not detected in the pre- and post-dredging sediment samples (Appendix 1).

### 3.3 Physicochemical analysis - phase 2

In the second phase, total suspended sediments (sand and silt) increased significantly (p = 0.007) in the dredge working area (DS and DD) in the first months of dredging (Appendix 2). Moreover, higher values (p = 0.008) are observed below the discharge site. Although then, at the downstream site (DDis) it normalizes, with values similar to those upstream of the Paraná River (except the site near the discharge of the Baradero River into the Paraná River) (Appendix 2). Suspended organic matter shows a similar pattern to total suspended sediment with lower but significant differences between upstream and downstream dredging sites (p = 0.009). While in the discharge zone, the values upstream and below the discharge point are similar, although it is observed that these at the discharge point were higher but not significant (p = 0.13) (Appendix 2).

**Fig. 5:**
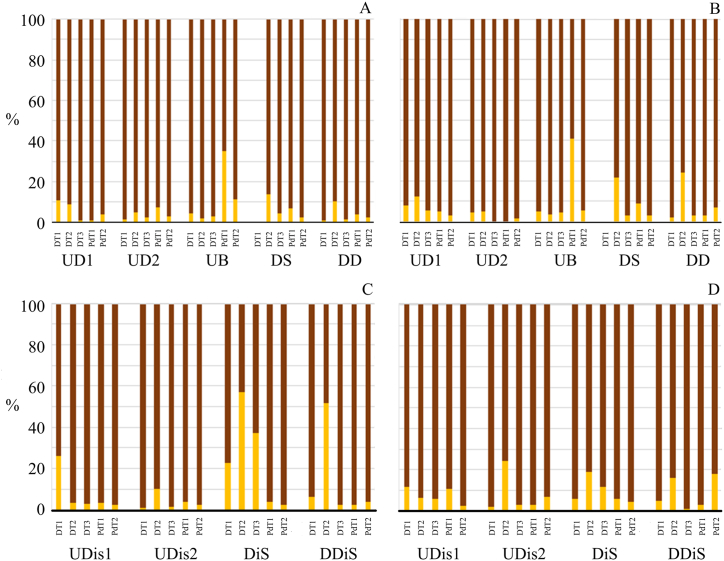
Percentage of very fine sand (column brown) (>62.5 μm) and fine sediment (column yellow) (silt and clay: <62.5 μm) in the sampling during the dredging, the discharge works and post-dredging (A: dredge area; C: discharge area). In addition, the organic material associated with very fine sand (columns brown) (>62.5 μm) and fine sediment (columns yellow) (silt and clay: <62.5 μm) (B: dredge area; D: discharge area), indicating the site and time of the sampling according to Table 1.

The suspended material at most dredging sites consisted of more than 80 % fine sediment (silt and clay: <62.5 μm) (Fig. 5). However, resuspension of very fine sand (>62.5 μm) is observed at the end of the dredging tasks in the samples downstream of the dredger (DS), the variation being significant (p = 0.03). A similar situation occurs in the discharge, with values of 40 % sand in the material deposited at the end of the dredging work. The percentage of organic matter in the sandy material is lower than in the fine sediment, with 75 % of the samples being greater than 90 % of organic matter at both the dredging and discharge sites (Fig. 5). Fine sediment, i.e. silt and clay, was most abundant in bottom samples during dredging and post-dredging at most sites. Only in a few samples were sand particles more abundant than fine sediment. Dredging did not significantly change the sand: silt: clay ratio in the dredged area (0.31:0.51:0.18) and the discharge area (0.33:0.44:0.23) (p = 0.85) (Appendix 2).

The pH values were similar at all sites (p = 0.089) related to dredging and discharge operations. It was somewhat more basic in the access channel, maneuvering area, and proximity to the city (7.8 ± 0.25) and slightly close to neutral values in the main channel (7.6 ± 0.16). Furthermore, after dredging operations, the values were similar (7.9 ± 0.28 for the dredging area and 7.6 ± 0.07 for the main channel of Paraná River) (p = 0.052).

**Fig. 6:**
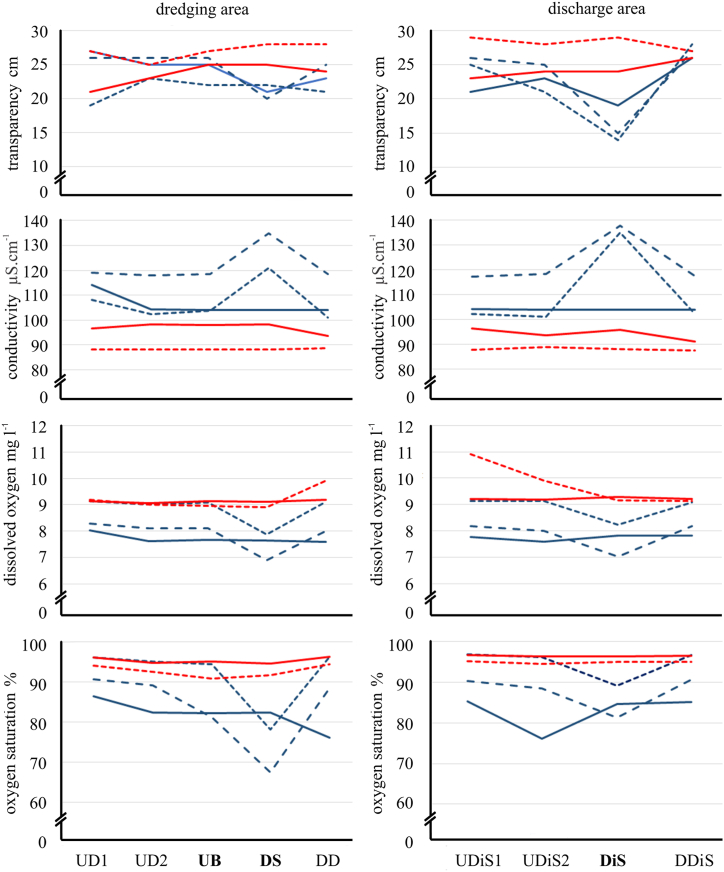
Some limnological parameters measured during and after the dredging procedure at sampling sites and times. Blue line during dredging actions and red line in post-dredging (dredge sampling: blue full line: 05/10/2023, blue dot line: 06/08/2023, blue dot line: 07/06/2023; post-dredge sampling: red full line: 08/17/2023, red dot line: 09/19/2023), indicating the sampling site according to Table 1 (bold letter: ship position).

The other parameters measured (e.g. transparency, conductivity, ClNa, dissolved oxygen, and its percentage of saturation) show significant variation in the samples downstream of the dredger, both in the dredging area and in the discharge area (p < 0.001) (Fig. 6). After the dredging tasks, the values were normalized, showing similar measurements between upstream and downstream of the dredging or discharge zones (p = 0.205) (Fig. 6).

### 3.4 Bacteriological analysis

**Fig. 7:**
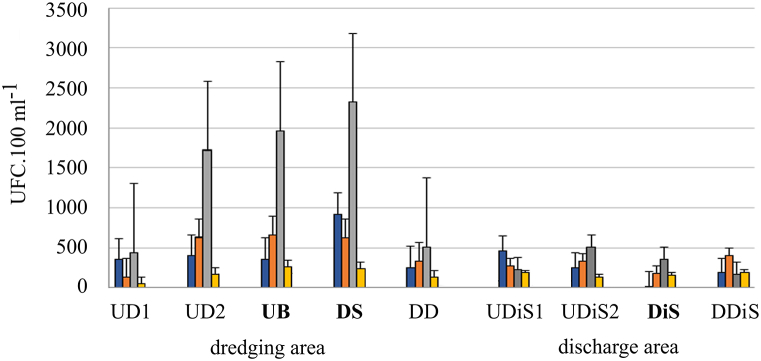
Mean and standard deviation of UFC values for *Escherichia coli* during the study (Dredging period: Blue. Red and grey columns; Post dredging: yellow column), indicating the sampling site according to Table 1 (bold letter ship position). On the third sampling day (grey column) an intense storm (34 mm) occurred.

Higher values of *E*. *coli* were observed in samples downstream of the dredge (stern) when it was dredging works, and the values increased significantly after rainfall events (p = 0.009) (Fig. 7). Post-dredging samples had lower and statistically different colony forming unit values (p = 0.049). In the discharge area, higher values were observed during the discharge times, and in the later samples, they were lower (Fig. 7).

**Fig. 8:**
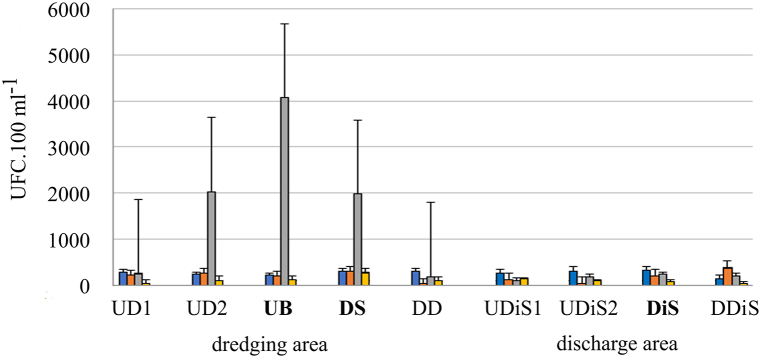
Mean and standard deviation of UFC values for *Enterococcus* during the study (Dredging period: Blue. Red and grey columns; Post dredging: yellow column), indicating the sampling site according to Table 1 (bold letter: ship position). On the third sampling day (grey column) an intense storm (34 mm) occurred.

Like *E*. *coli*, *Enterococcus* bacteria increased significantly in samples after rainfall events in the dredging area (p = 0.008). In the rest of the samples, the values of colony forming units were similar to those of the discharge zone (Fig. 8).

**Fig. 9:**
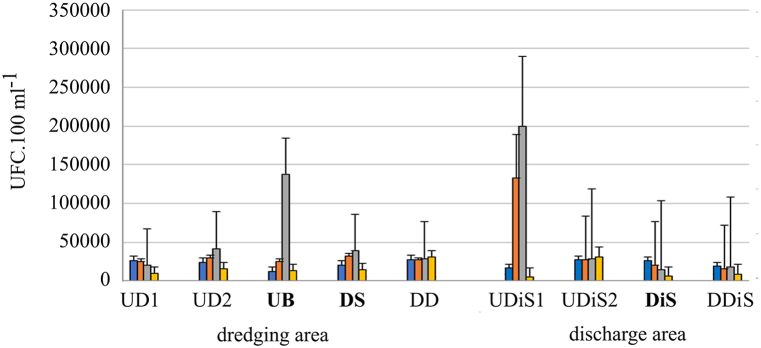
Mean and standard deviation of UFC values for Total Coliform during the study (Dredging period: Blue. red and grey columns; Post dredging: yellow column), indicating the sampling site according to Table 1 (bold letter: ship position). On the third sampling day (grey column) an intense storm (34 mm) occurred.

At the beginning of the dredging work, TC (total coliform) bacteria presented similar values of colony forming units in the samples. While in the third sampling, which corresponded to moments of intense rainfall events (80 mm), it had significantly higher values, mainly upstream of the dredge (bow) (Fig. 9). This sample corresponded to the time of dredging in the dock port area. After dredging, the values decreased with lower values at the time of dredging although these did not show differences between the samples (p = 0.09). A similar variation occurred in the discharge area with significant increases during sampling coinciding with rainfall events (p = 0.036) (Fig. 9).

**Fig. 10:**
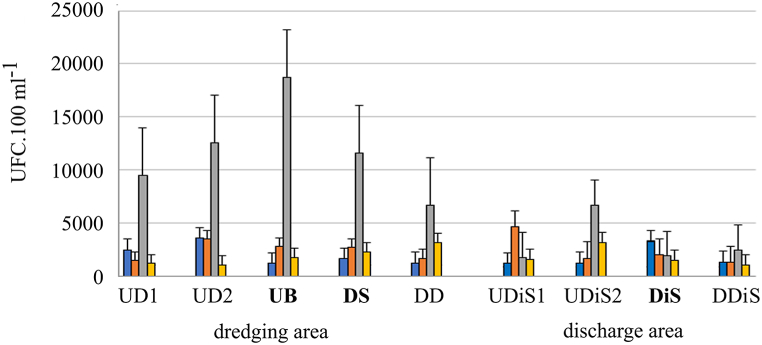
Mean and standard deviation of UFC values for Heterotrophic during study (Dredging period: Blue. red, grey columns; Post dredging: yellow column), indicating the sampling site according to Table 1 (bold letter: ship position). On the third sampling day (grey column) an intense storm (34 mm) occurred.

The Het group showed a similar relationship to the rest (Fig. 10), with a significant increase in those samples from the dredging area taken during rainfall events (p = 0.013).

## 4 Discussion

Dredging has been considered one of the necessary and controversial activities that man must carry out so that ports can function, and products can be shipped and unloaded to different regions of the world. This entails economic costs and risks to the health of aquatic environments, potential effects on aquatic organisms, water quality, and land management [42].

The Paraná River with its shallow lakes and secondary rivers that make up the floodplain, a mosaic of aquatic environments, is a eutrophic and very turbid ecosystem. The waters of the Paraná River carry a large amount of sediments that come mainly from the Andes mountain range with maximums of 1158 mg L^−1^ [24]. One of the most important tasks in ports located along the river is to maintain the depth by dredging within values that allow ships to enter and leave the port without difficulty. This is a problem because the natural turbidity of the river, due to the amount of suspended sediment, makes it impossible to control the depth using satellite data as is currently the case in other ports around the world [43]. In the Paraná, routine bathymetry is used to estimate the magnitude of sedimentation to ensure safe maneuverability and navigability in the access channels and dock area.

One consideration to keep in mind is that dredging simplifies the bottom morphology that was built by natural forces over time [44]. This homogenization of the environment modifies the habitat [45], beginning its natural reconstruction when the dredging is finished. It is necessary to maintain the macro-shape of the bottom with its thalweg to avoid changes in river flows and variations in negative sedimentation and erosion rates. Erosion and sedimentation are not continuous and uniform over seasons and years and are due to variations in rainfall and flow velocity [46]. Currently, sedimentation and erosion rates, caudal, and flow velocity should be considered as more important variables than in previous years. This is due to the effect of global warming and the greater recurrence and intensity of the El Niño- La Niña phenomenon [47], which confirms the need for continuum monitoring of its ecological implications.

The discharge of dredged sediments into the main channel, in areas with depths close to 30 m and velocities varying from 0.44 to 1.11 m s^−1^ [48], does not significantly modify the shapes of the river bed since the fine material sediments slowly and has time to distribute in the space of the river bottom. Moreover, the sediment transport capacity of the river is responsible for the morphological changes induced by erosion and deposition rates. It is evident from the analyses of velocities and accumulated erosion and deposition that zones with higher velocity transport more sediment than the other zones [49].

Following dredging activities, the accumulation of all substances present in the area and associated with land use in the region begins in the bed sediment. However, during dredging, chemical desorption of particulate material to the dissolved phase occurs due to resuspension of the bed sediment, but this requires time and certain conditions. This is key in determining the chemical impact that may occur in the water column due to dredging. The time, from a few hours to weeks and months, required for desorption is due to chemical partitioning factors. It includes the chemical substances and their physicochemical characteristics, their concentration and interaction with other chemical substances, particle geometry, pH, salinity and organic carbon content of the sediment [50]. Time is a key factor when considering the presence or loss of particulate matter in the sediment or its entrapment. Therefore, some contaminants in the particulate fraction resuspended by dredging could not have time to be desorbed and it is resettled in the sediment where they are discharged.

Among the desorbed chemicals, metals are important to analyze the effects of dredging and what fraction of them is bioavailable. Metals bound to sediment particles have coprecipitated or removed by oxyhydroxides and iron/manganese carbonates associated with organic matter. Only a fraction is available under normal conditions. While in deeper and anoxic sediments, oxyhydroxides dissolve but metals are captured by sulfides, formed by sulfate reduction [51], inaccessible to biological communities [52]. The transition zone between the oxygenated and anoxic sections is a thin zone where there are no oxyhydroxide or sulfide phases. Some metals are solubilized in this section, it is the first to be dredged and presents the greatest immediate risks. Dredging has been mainly related to remobilized metals associated with sediment particles in the water column, which change their environmental conditions and promote the change of metals from the particles to the dissolved state [53,54].

Another situation is the Fe oxidation, which also causes the precipitation of iron (oxo)hydroxides [55]. It forms a very strong adsorbent layer on the surface of the newly dredged bottom and reduces the release of metals [[56], [57], [58]]. The process could take some time depending on environmental conditions, e.g. concentration, pH, organic material, among others.

Although the release of total metals can be large and greater than the risk levels indicated in recommendations [59,60], the concentrations of bioavailable metals are generally lower, and this may or may not produce direct toxicities as in other environments [61]. However, these concentrations can contribute to promoting antibiotic resistance in different groups of bacteria [62]. This occurs through the selection of resistant strains, co-selection of resistance genes, and facilitation of the horizontal gene transfer, generating potential risks to public health [63,64].

Furthermore, sediment resuspension during dredging promotes the release of nutrients. There are complex interactions between each type of nutrient and the nature of particulate material bound by cohesive force and residence time in the water column [65]. Nutrient release to the overlying water is affected by the concentration gradient, hydrodynamic variability, temperature, pH, and bioturbation, which cause internal release under different static and/or dynamic conditions of the environment. Among the most significant conditions is gradient diffusion as the main pathway of nutrient release [66]. Temperature affects nutrient release when the factor increases due to seasonal change. On the one hand, the rate of release of chemical substances is modified, and, on the other hand, microbial stimulation happens for the transformation of organic elements into inorganic substances [67,68]. The latter has a certain delay due to the time needed by the microbiota to adapt to the new condition.

Dredging with removal of the surface layer and resuspension of the sediment modifies the physicochemical conditions of the bottom generated since the last dredging, acidifying the sediment and the water column [69]. The decomposition of organic matter and with it the release of hydrogen ions are responsible for this change. Decreased oxygen is also associated with higher suspended solid values, turbidity or decreased transparency, which limits light penetration, and could cause a decrease in photosynthetic activity [70,71]. Lotic systems, such as the Baradero and Paraná rivers with a high concentration of suspended particulate matter and constant movement of water, would not represent a significant factor in the reduced light penetration. However, it represents one more aspect in the processes that occur during dredging, along with the effects caused by rainfall. Turbidity and total suspended solids (i.e. suspended inorganic solids and suspended organic solids) increased after heavy rainfall events, which is associated with increased freshwater inflow carrying particulate matter from the edges.

Furthermore, nutrients such as nitrate and ammonium could come from wastewater effluents and upstream nitrification processes associated with urban and agricultural areas [72]. Ammonium, depending on the pH, could be found in its non-ionized form (ammonia [NH_3_]), considered toxic to fish [73]. The pH changes regarded during dredging could increase the proportion of NH_3_, and non-ionized ammonia values could show toxic levels for biological communities [74]. The concentration of phosphate, whose increase is usually attributed to fertilizers via field and wastewater [75], has not yet caused eutrophication processes in the Baradero and Paraná Rivers. The amount of nutrients corresponds to the deposition processes that occurred mainly in the dock area since the last dredging work. This is due to the discharge of rainwater and wastewater from the city since the waters of the Paraná and Baradero rivers present very low concentrations or below the detection limit.

In addition, nutrient deposits accumulated since the last dredging, mainly from the city, port activities and agricultural land, were resuspended in the water column. In this way, nutrients based on nitrates and total phosphorus become available together with the solids, increasing the suspended and dissolved material in the water column.

Several decades ago, studies referred to the presence of soluble organic contaminants available in the water column after dredging [[76], [77], [78]]. However, what was observed in this work is that these contaminants must be at a very low concentration and/or the dilution of the Baradero and Paraná rivers led to these values being below the detection limit. Similar results have been observed for PAHs measured during dredging works. The soluble phases of petroleum derivatives and chlorinated hydrocarbons associated with the sediment are possibly below the detection limit, with minimal resuspensions occurring during dredging activity, as indicated in other work [79]. The release of these compounds from the sediment requires certain combinations of pH, oxidation-reduction potential, and time that have not been achieved in this dredging and discharge process.

The dredging was carried out during the dry phase or low level of the Paraná River, so the water flow was lower. The reduction in flow during the low water period makes water masses more exposed to the action of the wind [80,81]. In addition to the direct effect of the dredging process in general, the shallower depth, and longer residence time of water in an area could provoke the resuspension of sediments and associated organic matter, facilitating the increase and prevalence of bacterial concentrations. The significant increase in the concentration of different groups of bacteria, associated with rainfall events, possibly occurs due to the drag of solids and liquids from the city (contributed by stormwater discharges) and runoff from the floodplain. It includes soils with agricultural and livestock activity in surrounding areas [82], in concurrence with the increase in turbidity, total suspended solids, suspended inorganic solids and suspended organic solids. Likewise, when precipitation is abundant, it increases turbulence and facilitates the resuspension of sediments and bacteria along with them [83].

The decrease in bacterial concentrations towards the last sampling, where no rainfall events were recorded and water and sediment movements ceased due to dredging, showed the normalization and recovery of the system. *Enterococcus* concentrations in sediments have been shown to increase in response to discharges from sources including wastewater treatment plants, streams, and stormwater [[84], [85], [86]]. *Enterococcus* from soil, wastewater, and dog feces was also tested in freshwater mesocosms [87] with similar decay rates [88], and without affecting the inactivation rate [89]. Recently, a study comparing the decay of *Enterococcus* from sewage effluent or composite cow manure samples found that enterococci from cow manure persisted significantly longer in freshwater than those from sewage [90].

For several decades it has been indicated that the different physicochemical parameters and bacteriological changes generated by dredging do not extend very far downstream [91]. This should be noted that in this study they do not remain for a long time, and 2 km downstream of the dredging discharge in the Paraná River, values similar to those that characterize this river are observed. Furthermore, after 60 days of dredging, the system was completely normalized, including both the dredged and discharge areas.

## 5 Conclusion

The port and dredging area are located south of the city, near of sewage drains. Moreover, upstream there are nautical clubs and tourist ventures. The 1.5 km intervention on a river of just under 5,000 km has a significant impact that punctually affects the river's landscape and its functioning. However, it could represent a situation of heterogeneity in space and time of the habitats present in this stretch of river if the time elapsed until the next dredging works allowed the development of the habitats that characterize this stretch.

The proximity of storm drains, sewers and wastewater makes the port's maneuvering area and the access channel a risk area. This is due to the accumulation of nutrients since these will be associated with fine sediments and have electrical charges that allow the coupling of molecules containing N and P. Then, they are released, modifying the energy capacity of the place.

The increase in nutrients must be monitored and related to the possible risks of eutrophication processes that could occur downstream and thus the possibility of the appearance of cyanophycean blooms.

Dredging activities cause disturbances in aquatic ecosystems that can affect the metabolism of aquatic species, so it is concluded that although dredging is beneficial in socioeconomic terms, its environmental effects require adequate management to avoid environmental problems and diseases and to have good environmental safety.

With strategic planning, dredging can be carried out with the premise of preserving aquatic environments, their margins and the associated urban environment. The effects of dredging activity can be minimized by combining management measures and knowledge of the effects on species.

## CRediT authorship contribution statement

**Maria Josefina Gonzalez:** Writing – review & editing, Resources, Methodology, Investigation, Data curation. **Stella Maris Gonzalez:** Writing – review & editing, Validation, Supervision, Methodology, Formal analysis, Data curation. **Aldo Raul Paira:** Writing – review & editing, Supervision, Methodology, Investigation, Formal analysis, Conceptualization. **Pablo Agustín Collins:** Writing – original draft, Supervision, Software, Resources, Project administration, Methodology, Investigation, Funding acquisition, Formal analysis, Data curation, Conceptualization.

## Data availability statement

Data are available in Appendix A, Appendix A as Supplementary material.

## Declaration of Competing Interest

The authors declare the following financial interests/personal relationships which may be considered as potential competing interests: Pablo Collins reports financial support and administrative support were provided by Santa Fe Agency of Sciences ASACTEI 23525874. Pablo Collins reports financial support and equipment, drugs, or supplies were provided by 10.13039/501100002923CONICET Santa Fe. Pablo Collins reports a relationship with CONICET Santa Fe that includes: board membership and employment. Pablo Collins has patent not correspond pending to not correspond. The authors declare that they have no known competing financial interests or personal relationships that could have appeared to influence the work reported in this paper. If there are other authors, they declare that they have no known competing financial interests or personal relationships that could have appeared to influence the work reported in this paper.

## Footnotes

Appendix A Supplementary data to this article can be found online at https://doi.org/10.1016/j.heliyon.2024.e41224.

## References

[1] Zhang S., Zhou Q., Xu D., Lin J., Cheng S., Wu Z. (2010). Effects of sediment dredging on water quality and zooplankton community structure in a shallow of eutrophic lake. J. Environ. Sci..

[2] Vammen K., Vaux H., Roldán G., González E., Tundisi J., Izurieta R., Fabrega J. (2019).

[3] Mateo-Sagasta J., Zadeh S.M., Turral H., Burke J. (2017). Food and Agriculture Organization of the United Nations, Rome and the International Water Management Institute on Behalf of the Water Land and Ecosystems Research Program.

[4] José de Paggi S., Paggi J.C., Collins P., Collins J., Bernal G. (2008). Water quality and Zooplankton composition in a receiving pond of the stormwater runoff from an urban catchment. J. Environ. Biol..

[5] Laetz C.A., Hecht S.A., Incardona J.P., Collier T.K., Scholz N.L., Triquet C.A., Amiard J.C., Mouneyrac C. (2015). Aquatic Ecotoxicology: Advancing Tools for Dealing with Emerging Risks.

[6] Li C., Zheng X., Zhao F., Wang X., Cai Y., Zhang N. (2017). Effects of urban non-point source pollution from baoding city on baiyangdian lake. China, Water.

[7] WHO (2003).

[8] Magaña-Arachchi D.N., Wanigatunge R.P., Prasad M.N.V., Grobelak A. (2020). Waterborne Pathogens: Detection and Treatment.

[9] Stec J., Kosikowska U., Mendrycka M., Stępień-Pyśniak D., Niedźwiedzka-Rystwej P., Bębnowska D., Hrynkiewicz R., Ziętara-Wysocka J., Grywalska E. (2022). Opportunistic pathogens of recreational waters with emphasis on antimicrobial resistance—a possible subject of human health concern. Int. J. Environ. Res. Public Health.

[10] Ke X., Gui S., Huang H., Zhang H., Wang C., Guo W. (2017). Ecological risk assessment and source identification for heavy metals in surface sediment from the Liaohe River protected area, China. Chemosphere.

[11] Durak J., Rokoszak T., Skiba A., Furman P., Styszko K. (2021). Environmental risk assessment of priority biocidal substances on Polish surface water sample. Environ. Sci. Pollut. Res..

[12] Digué T.M., Tinda D., Bertrand N.G., Salomon M.B., Dikdim D.J.-M., Mianpereum T. (2023). Contamination and potential risks of heavy metals in the sediments of the chari and logon rivers in N'djamena, Chad, open J. Soil Sci..

[13] Murali Krishna I.V., Manickam V., Murali Krishna I.V., Manickam V. (2017). Environmental Management Science and Engineering for Industry.

[14] Kowalkowski T., Gadzała-Kopciuch M., Kosobucki P., Krupczyńska K., Ligor T., Buszewski B. (2007). Organic and inorganic pollution of the Vistula River basin. J. Environ. Sci. Health - Toxic/Hazard. Subst. Environ. Eng..

[15] Montuori P., De Rosa E., Sarnacchiaro P., Di Duca F., Provisiero D.P., Nardone A., Triassi M. (2020). Polychlorinated biphenyls and organochlorine pesticides in water and sediment from Volturno River, Southern Italy: occurrence, distribution and risk assessment. Environ. Sci. Eur..

[16] Zhihao W., Xia J., Shuhang W., Li Z., Lixin J., Junyi C., Qing C., Kun W., Cheng Y. (2021). Mobilization and geochemistry of nutrients in sediment evaluated by diffusive gradients in thin films: significance for lake management. J. Environ. Manag..

[17] Borgnino L., Orona C., Avena M., Maine M.A., Rodríguez A., De Pauli C.P. (2006). Phosphate concentration and association as revealed by sequential extraction and microprobe analysis: the case of sediments from two Argentinean reservoirs. Water Resour. Res..

[18] Wakeham S.G., Canuel E.A., Lerberg E.J., Mason P., Sampere T.P., Bianchi T.S. (2009). Partitioning of organic matter in continental margin sediments among density fractions. Mar. Chem..

[19] Gibson B.D., Ptacek C.J., Blowes D.W., Daugherty S.D. (2015). Sediment resuspension under variable geochemical conditions and implications for contaminant release. J. Soils Sediments.

[20] Alekseevskiy N.I., Berkovich K.M., Chalov R.S. (2008). Erosion, sediment transportation and accumulation in rivers. Int. J. Sediment Res..

[21] Jungen H., Specht P., Ovens J., Lemper B., Freitag M., Kotzab H., Megow N. (2021). Dynamics in Logistics.

[22] Bray R.N. (2008).

[23] Pachepsky Y.A., Shelton D.R. (2011). *Escherichia coli* and fecal coliforms in freshwater and estuarine sediments. Crit. Rev. Environ. Sci. Technol..

[24] Metcalfe C.D., Menone M., Collins P., Tundisi J. (2020).

[25] Collins P., Marchese M., Metcalfe C.D., Menone M., Collins P., Tundisi J. (2020). The Paraná River Basin: Protecting Ecosystem Services through Effective Water Management, Earthscan from Routledge.

[26] Rocha P.C., Leal A.C., de Araujo R.R., Di Mauro C.A., Ribeiro W.C., Mehmood H., Metcalfe C.D., Menone M., Collins P., Tundisi J. (2020). The Paraná River Basin: Protecting Ecosystem Services through Effective Water Management. Earthscan from Routledge.

[27] Haynes D., Johnson J.E. (2000). Organochlorine, heavy metal and polyaromatic hydrocarbon pollutant concentrations in the great barrier reef (Australia) environment: a review. Mar. Pollut. Bull..

[28] Reine K.J., Dickerson D.D., Clarke D.G. (1998).

[29] Reine K.J., Clarke D., Dickerson C. (2014). Characterization of underwater sounds produced by hydraulic and mechanical dredging operations. J. Acoust. Soc. Am..

[30] Iriondo M.H., Paggi J.C., Parma M.J. (2007).

[31] Vanoni V.A. (2006). Sedimentation engineering: Classic edition, MOP54.

[32] Bouyoucos G.L. (1962). Hydrometer method improved for making particle size analysis of soils. Agron. J..

[33] APHA (1998).

[34] EPA (U.S. Environmental Protection Agency) (2007).

[35] EPA (U.S. Environmental Protection Agency) (2014).

[36] EPA (U.S. Environmental Protection Agency) (2024). https://www.epa.gov/cwa-methods.

[37] APHA (2017).

[38] European Union (EU) (2006).

[39] EPA (U.S. Environmental Protection Agency) (2015). FAQ: NPDES water-quality based permit limits for recreational water quality criteria physical parameters on the in-situ survival of *Escherichia coli* MC-6 in an estuarine environment. Appl. Environ. Microbiol..

[41] Hammer O., Harper D.A.T., Ryan P.D. (2001). PAST, Paleontological Statistics software package for education and data analysis. Palaeontol. Electron..

[40] Zar J.H. (1996).

[42] Snyder G. (1976). Effects of dredging on aquatic organisms with special application to areas adjacent to the Northeastern Pacific Ocean. Mar. Fish. Rev..

[43] Mateo-Pérez V., Corral-Bobadilla M., Ortega-Fernández F., Rodríguez-Montequín V. (2021). Analysis of the spatio-temporal evolution of dredging from satellite images: a case study in the Principality of Asturias (Spain). J. Mar. Sci. Eng..

[44] Rahman M.M., Hasan M.S., Eusufzai M.K., Rahman M.M. (2021). Impacts of dredging on fluvial geomorphology in the Jamuna River, Bangladesh. J. Geosci. Environ. Prot..

[45] Smith W.S., da Silva F.L., Biagioni R.C. (2019). River dredging: when the public power ignores the causes, biodiversity and science. Ambient. Soc..

[46] Islam K., Al Kibriya N., Dustegir M. (2018). Impact analysis of sand dredging from alluvial tidal river. E3S Web of Conferences.

[47] Torres M.V., Giri F., Collins P.A. (2016). "La Niña" phenomenon on the relationship between decapod populations and fishes in temporarily isolated shallow lakes. Mar. Fresh. Res..

[48] Paira A.R., Drago E.C., Iriondo M.H., Paggi J.C., Parma M.J. (2007). The Middle Paraná River: Limnology of a Subtropical Wetland.

[49] Rahman A., Yunus A. (2016). Hydrodynamic and morphological response to dredging: analysis on gorai river of Bangladesh. Int. J. Innov. Res. Sci. Eng. Technol..

[50] Tomson M.B., Kan A.T., Chen W., Hunter M.A. (2003). Hazardous Substance Research Center, South/Southwest.

[51] Fakhraee M., Li J., Katsev S. (2017). Significant role of organic sulfur in supporting sedimentary sulfate reduction in low-sulfate environments. Geochim. Cosmochim. Ac..

[52] Stockdale A., Davison W., Zhang H. (2010). Formation of iron sulfide at faecal pellets and other microniches within suboxic surface sediment. Geochim. Cosmochim. Ac..

[53] Van den Berg G.A., Meijers G.G.A., Van Der Heijdt L.M., Zwolsman J.J.G. (2001). Dredging related mobilisation of trace metals: a case study in The Netherlands. Water Res..

[54] Chen X., Wang Y., Sun T., Huang Y., Chen Y., Zhang M., Ye C. (2021). Effects of sediment dredging on nutrient release and eutrophication in the gate-controlled estuary of Northern Taihu Lake. J. Chem..

[55] Dang D.H., Layglon N., Ferretto N., Omanović D., Mullot J.U., Lenoble V., Mounier S., Garnier C. (2020). Kinetic processes of copper and lead remobilization during sediment resuspension of marine polluted sediments. Sci. Total Environ..

[56] Goossens H., Zwolsman J.J.G. (1996). An evaluation of the behaviour of pollutants during dredging activities. Terra Aqua (Engl. Ed.).

[57] Eggleton J., Thomas K.V. (2004). A review of factors affecting the release and bioavailability of contaminants during sediment disturbance events. Environ. Int..

[58] Roberts D.A. (2012). Causes and ecological effects of resuspended contaminated sediments (RCS) in marine environments. Environ. Int..

[59] EEC (2008). Directive 2008/105/EC of the European Parliament and of the Council of 16 December 2008 on environmental quality standards in the field of water policy, amending and subsequently repealing Council Directives 82/176/EEC, 83/513/EEC, 84/156/EEC, 84/491/EEC, 86/280/EEC and amending Directive 2000/60/EC of the European Parliament and of the Council. Off. J. Eur. Union.

[60] EPA (U.S. Environmental Protection Agency) (2010). http://water.epa.gov/scite.ch/swguidance/standards/criteria/current/index.cfm.

[61] Miró J.M., Megina C., Donázar-Aramendía I., García-Gómez J.C. (2022). Effects of maintenance dredging on the macrofauna of the water column in a turbid estuary. Sci. Total Environ..

[62] Fernandes P., Ferreir B.S., Cabral J.M. (2003). Solvent tolerance in bacteria: role of efflux pumps and cross-resistance with antibiotics. Int. J. Antimicrob. Agents.

[63] Rilstone V., Vignale L., Craddock J., Cushing A., Filion Y., Champagne P. (2021). The role of antibiotics and heavy metals on the development, promotion, and dissemination of antimicrobial resistance in drinking water biofilms. Chemosphere.

[64] Friedman M. (2015). Antibiotic-resistant bacteria: prevalence in food and inactivation by food-compatible compounds and plant extracts. J. Agric. Food Chem..

[65] Pastor A., Larsen J., Mohn C., Saurel C., Petersen J.K., Maar M. (2020). Sediment transport model quantifies plume length and light conditions from mussel dredging. Front. Mar. Sci..

[66] Li W., Xu S., Chen X., Han D., Mu B. (2023). Influencing factors and nutrient release from sediments in the water level fluctuation zone of biliuhe reservoir, a drinking water reservoir. Water.

[67] Hu K.S., Bouchard V., Moore R.H. (2008). Factors affecting denitrification in agricultural headwater streams in Northeast Ohio, USA. Hydrobiologia.

[68] Van Kessel M.A.H.J., Speth D.R., Albertsen M., Nielsen P.H., Op den Camp H.J.M., Kartal B., Jetten M.S.M., Lücker S. (2015). Complete nitrification by a single microorganism. Nature.

[69] Jones-Lee A., Lee G.F. (2005). Water Encyclopedia.

[70] Descy J.-P., Reynolds C.S., Padisak J. (1994). Proceedings of the 9th Workshop of the International Association of Phytoplankton Taxonomy and Ecology (IAP) Held in Mont Rigi (Belgium).

[71] Nunes P., Roland F., Amado A.M., Resende N.S., Cardoso S.J. (2022). Responses of phytoplanktonic chlorophyll-a composition to inorganic turbidity caused by mine tailings. Front. Environ. Sci..

[72] Mendiguchía C., Moreno C., García M. (2007). Evaluation of natural and anthropogenic influences on the Guadalquivir River (Spain) by dissolved heavy metals and nutrients. Chemosphere.

[73] Brinkman S.F., Woodling J.D., Vajda A.M., Norris D.O. (2009). Chronic toxicity of ammonia to early life stage rainbow trout. Trans. Am. Fish. Soc..

[74] Thea M.E., Puglis H.J., Kent D.B., López Durán J., Bradshaw L.M., Farag A.M. (2024). Ammonia and aquatic ecosystems – a review of global sources, biogeochemical cycling, and effects on fish. Sci. Total Environ..

[75] Mainstone C.P., Parr W. (2002). Phosphorus in rivers - ecology and management. Sci. Total Environ..

[76] Thomann R.V. (1989). Bioconcentration model of organic chemical distribution in aquatic food chains. Environ. Sci. Technol..

[77] McAnally W.H., Friedrichs C., Hamilton D., Hayter E., Shrestha P., Rodriguez H., Sheremet A., Teeter A. (2007). Management of fluid mud in estuaries, bays, and lakes. I. Present state of understanding on character and behavior. J. Hydraulic. Eng..

[78] Bridges T.C., Gustavson K.E., Schroeder P., Ells S.J., Hayes D., Nadeau S.C., Palermo M.R., Patmont C. (2010). Dredging processes and remedy effectiveness: relationship to the 4 Rs of environmental dredging. Integr. Environ. Assess. Manag..

[79] Herbich J.B. (2000).

[80] Carignan R., Planas D. (1994). Recognition of nutrient and light limitation in turbid mixed layers: three approaches compared in the Paraná floodplain (Argentina). Limnol. Oceanogr..

[81] Izaguirre I., O'Farrell I., Tell G. (2001). Variation in phytoplankton composition and limnological features in a water–water ecotone of the Lower Paraná Basin (Argentina). Freshwat. Biol..

[82] Heathwaite A.L., Bieroza M. (2021). Fingerprinting hydrological and biogeochemical drivers of freshwater quality. Hydrol. Process..

[83] Rossi A., Wolde B.T., Lee L.H., Wu M. (2020). Prediction of recreational water safety using *Escherichia coli* as an indicator: case study of the Passaic and Pompton rivers, New Jersey. Sci. Total Environ..

[84] Obiri-Danso K., Jones K. (2000). Intertidal sediments as reservoirs for hippurate negative campylobacters, salmonellae and faecal indicators in three EU recognised bathing waters in North West England. Water Res..

[85] Le Fevre N.M., Lewis G.D. (2003). The role of resuspension in enterococci distribution in water at an urban beach. Water Sci. Technol..

[86] Ferguson D.M., Moore D.F., Getrich M.A., Zhowandai M.H. (2005). Enumeration and speciation of enterococci found in marine and intertidal sediments and coastal water in southern California. J. Appl. Microbiol..

[87] Anderson K.L., Whitlock J.E., Harwood V.J. (2005). Persistence and differential survival of fecal indicator bacteria in subtropical waters and sediments. Appl. Environ. Microbiol..

[88] Staley C., Dunny G.M., Sadowsky M.J., Sariaslani S. (2014).

[89] Noble R.T., Lee I.M., Schiff K.C. (2004). Inactivation of indicator micro-organisms from various sources of faecal contamination in seawater and freshwater. J. Appl. Microb..

[90] Korajkic A., McMinn B.R., Harwood V.J., Shanks O.C., Fout G.S., Ashbolt N.J. (2013). Differential decay of enterococci and *Escherichia coli* originating from two fecal pollution sources. Appl. Environ. Microbiol..

[91] Grimes D.J. (1980). Bacteriological water quality effects of hydraulically dredging contaminated upper Mississippi River Bottom Sediment. Appl. Environ. Microbiol..

